# Foundational issues of network models in biology

**DOI:** 10.1007/s00422-026-01044-6

**Published:** 2026-06-09

**Authors:** Danilo Bondi, Damiano Bondi

**Affiliations:** 1 Department of Neuroscience, Imaging and Clinical Sciences, University “G. d’Annunzio” Chieti—Pescara, Via Dei Vestini, 31, 66100 Chieti, Italy; 2 Department of Economics, Society & Politics, University of Urbino Carlo Bo, Urbino, Italy

**Keywords:** Graph, Complex adaptive systems, Relation, Epistemology, Person

## Abstract

Despite the growing prevalence of network models in biological and medical research, the philosophical foundations of these constructs remain elusive and insufficiently examined. Building on data-driven insights in systems biology and teleological models of integrative physiology, we have criticised agentless theory, relational ontologies, and cybernetic perspectives in biological contexts. We have posited the necessity for a philosophical advocacy of a holistic approach to biology alongside a relational epistemology. A foundational issue in network models in biology and physiology is recognising the network as the predominant meme of contemporary society, which has permeated all aspects of human life and has been cultivated within biology and physiology through modern theoretical constructs and practical applications. By discussing minimal cognition, tinkering, and stigmergy, we argued for a philosophical advocacy of the person as a relational cybernetic organism. Philosophical arguments concerning graph and relational models in biology can be resolved by embracing epistemic humility.

## Introduction

In its simplest form, a network consists of nodes and edges. Networks have been described in relation to nature, information, society, and technology. The field of network science has its roots in graph theory, a branch of mathematics first developed by the Swiss mathematician Leonhard Euler in 1735. In that lecture, Euler presented a solution to a bridge-crossing problem by using the geometry of position, marking the start of a subject that has since fascinated brilliant minds across all fields (Wilson 2013).

The abundance of large databases in fields like biology and information technology has driven the growth of network science, which focuses on analysing the structure of connections among the components of complex systems. Nurtured by evidence of scale-free networks in biology, the study of network effects has expanded and permeated all aspects of biological and medical research. Indeed, as Albert-László Barabási pointed out, «*networks pervade all aspects of human health*», including technological, social, and biological systems, which can be designed by simple and quantifiable organising principles (Barabási 2007).

The diversity and pervasiveness of network models in the biological sciences result from the understanding that most processes occur as networks controlled by sensors, signals, and effectors, whose complexity is unparalleled outside biology. Historically, network models have been widely applied in biology to address the challenges posed by biological complexity. However, conceptual and methodological aspects of network-based approaches still require a programmatic foundation. Valuable efforts have been made to address foundational issues in network explanation and modelling, to discuss the exploratory and heuristic functions of network models, and to systematise levels and hierarchies in network organisation through cross-disciplinary work by biologists and philosophers (Kostić et al. 2020). In this context, according to the intention of the “topological graph theory”, Huneman argues that network architecture, i.e., topology, constrains the dynamics of what takes place in the network, thereby.

detaching topological explanations from the consideration of mechanisms proper to the systems under study (Huneman 2018). From a methodological perspective, network models have been extensively studied; for example, Rathkopf considers network science a powerful method for understanding interconnectedness and the properties that make a system complex, rather than a continuation of mechanistic analysis (Rathkopf 2018). Craver argued that «*whether network models explain, how they explain, and how much they explain […] must be answered by fixing an explanandum phenomenon, considering how the model is applied to a target, and deciding what sorts of variables and relations count as explanatory*» (Craver 2016). Network science provides a strong foundation to study biological complexity, but a strong foundation is needed to a proper use of network science in biology. Despite recent philosophical investigations of network theories, the very foundations of the existence and use of network models in biology remain elusive.

### Biology and physiology in philosophy

To avoid the risk of falling into a foundational issue, it is necessary to examine the theme that gives our manuscript its title more closely. Biology can be easily defined as the study of life and living entities, while the definition of physiology and its evolution is more challenging. Currently, The Physiological Society defines physiology as «*the branch of biology that aims to understand the mechanisms of living things, from the basis of cell function at the ionic and molecular level to the integrated behaviour of the whole body and the influence of the external environment*» (https://www.physoc.org/explore-physiology/what-is-physiology/).

Welch has thoroughly examined the origins and development of the etymon, concluding that «*in Greek, the expression “physiology” (φυσιoλoγία) denotes literally “discourse on nature.” The Latinized term “physiologia” emerged from Greco-Roman antiquity as an idiom designating the “study of nature” or “natural philosophy”; and people who studied nature, in any or all of its parts, were called “natural philosophers” or “physiologers”*» (Welch 2009). In fact, philosophy originated in Ancient Greece as physiology, i.e., as a study of Nature.

The very first works of Western philosophy are all devoted to the study of Nature (φύσις, *physis*), as testified by the title attributed to them: Anaximander, Empedocles, Heraclitus, Parmenides, and Zeno all wrote essays known as “On Nature” (Περὶ Φύσεως, *Perì Physeos*). A famous passage from Plato’s *Phaedo* proves that at that time the idea of ​​the existence of a tradition of research on nature (*physiología*), from Thales to Socrates, was widespread: «When I was young, Cebes, I was tremendously eager for the kind of wisdom which they call investigation of nature (*perì physeos historían*). I thought it was a glorious thing to know the causes of everything, why each thing comes into being and why it perishes and why it exists» (Plato, *Phaedo*, 96a).

Significantly, these two disciplines, philosophy and physiology, diverged and later reconverged over the centuries, both increasingly focusing on human beings rather than nonhuman nature. Physiology, in particular, has undergone a significant shift in meaning relative to its original sense. As Welch underscores, Claude Bernard articulated the enduring notion of the *milieu intérieur* as the internal conditions for maintaining the living state, thereby conceptualising an integrated, multilevel functional entity with inherent control mechanisms, i.e., a system.

One of the foundational issues in biology is its theoretical connection to contemporary physiology: how, and to what extent, does the understanding of human functioning inform theoretical models of living beings in general? In the language of biology, for example, we can observe that in the first chapter of *The Origin of Species*, Darwin introduces natural selection through the concept of artificial selection (of plants and animals) practised by farmers. This passage is traditionally seen as a rhetorical *ploy* by Darwin to gradually introduce a revolutionary theory to the minds of right-thinking people. But we could overcome this reductive interpretation, underlying that artificial selection, that is *human* selection, is an experiential model upon which natural selection is built analogically. Nature *acts* as human beings do: it selects living species according to its own logic; «when we compare the dray-horse and race-horse, the dromedary and camel, the various breeds of sheep […] we must, I think, look further than to mere variability. We cannot suppose that all the breeds were suddenly produced as perfect and as useful as we now see them. […] *The key is man’s power of cumulative selection.* […] *he* [i.e., man] *may be said to have made for himself useful breeds* […] I have called this principle, by which each slight variation, if useful, is preserved, by the term natural selection, *in order to mark its relation to man’s power of selection*.» (Darwin 1859). Alfred Russel Wallace was fully aware of the theoretical issues and ambiguities implied by the metaphorical and anthropomorphic notion of “natural selection” (see Letter from Wallace to Darwin 1866), but less aware of its heuristic power and of the theoretical overall question (which also arises from alternative formulations, such as “survival of the fittest”): can we truly do without using our self-awareness to understand what is different from us? And would it really be epistemologically right to do so (i.e., would it lead to better, more objective knowledge)? These philosophical issues also apply to the teleological thinking—a long-lasting theme in philosophy of biology, as discussed below—in which the human experience of “acting towards something” works as a hermeneutic tool to explain behaviours of non-human living systems, ending up conceiving the whole nature as a “giant human being”. The theoretical trajectory of James Lovelock’s theory, which we will discuss later, exemplifies this dynamics: it began as a biological hypothesis called “geophysiology” and ended up anthropomorphising and, finally, sacralising planet Earth, Gaia, endowed with quasi-divine features (Lovelock 1989, 2001).

## Networks and biology

Reductionist biology takes the pieces (organisms, tissues, cells, molecules) apart, but most biological characteristics arise from a complex web of interactions among the numerous constituents that contribute to the structure and function of living elements. Network biology tends to a broader picture thus emerging as «*a new language which allows the cell’s molecular makeup to be discussed as a network of interacting constituents, and to spot and quantify the interplay between behaviour, structure and function*»; moreover, network biologists highlighted the interlink between structure, topology, network usage, robustness and function (Barabási and Oltvai 2004). Systems biology is closely linked to network biology through the examination of dynamical biological changes to analyse and predict state transitions in biological systems (Kirschner 2005). Similarly, integrative physiology departs from conventional organ-specific medicine by emphasising whole-body function and its applications to human health and disease. This approach involves the interconnectivity of mechanisms and regulatory functions across all biological levels (i.e., molecules, cells, tissues, and organs) (Sieck 2017). However, from an epistemological perspective, the viewpoints of systems biology and integrative physiology differ profoundly: the former is data-driven (homeostasis as an emergent mechanistic fact), whereas the latter is concept-driven (homeostasis as a key purpose that drives bodily processes). Although in systems biology functions represent purposes, integrative physiology necessarily addresses teleology, positing that the challenge and purpose of body processes and adaptations are homeostasis (Goldstein 2019). To illustrate this argument, let’s look at a very simplified example of how blood glucose is controlled: if sensors detect an increase in blood glucose levels, pancreatic β cells release insulin, which serves as a hypoglycemic function; on the other hand, α cells release the hyperglycemic hormone glucagon when there is a drop in blood glucose. Recalling the above-mentioned definition of physiology, understanding this integrated behaviour depends on sensing blood glucose and the release of pancreatic hormones. Instead, the common interpretation of integrative physiology is predicated on the “purpose” of integrated systems, i.e., maintaining blood glucose homeostasis by secreting pancreatic hormones in response to changes in glucose concentration. The concept of goal-directedness, along with those of non-mechanistic explanation and self-determination, forms the core of the “biological agency perspective”; as recently noted by DiFrisco and Gawne, biological agency can be understood as an organism’s capacity for goal-directed activity and self-determination that are not entirely explained by underlying mechanisms or natural selection; Biological Agency explanations are grounded in the Extended Evolutionary Synthesis and seek to revive the Aristotelian view of teleology as real rather than mere appearance (DiFrisco and Gawne 2025). However, the same authors have challenged this approach by arguing that phenomena which biological agency aims to explain can be better understood through complex multiscale feedback mechanisms evolving under natural selection, aligning with the perspective of evolutionary developmental (evo-devo) biology (see Sect. 2.1).

Philosophically, integrative physiology can be seen as one of the latest resurgences of Aristotle’s explicative model of “final cause”. According to Aristotle, living beings exhibit specific, recurring patterns of behaviour and change; in other words, they tend towards something. He calls this principle *entelechia*, which means “following an inner purpose” (Ritter 1932). Since the scientific revolution of early modernity, the concept of final cause has been excluded from epistemology, at least in physics. However, in biology, it has long been used as an explanatory concept for certain behaviours and aspects of living beings, under various names and even in its minimal form. Kant recognises the necessity of employing “teleological judgments” in interpreting living systems, as opposed to non-living ones (Zammito 2006; Quarfood 2006). Even within Darwinism, which can be seen as an anti-teleological paradigm of biological evolution, reproduction functions as a “key purpose driving biological processes” (Woodfield 1973; Lennox 1993; Depew 2008).

The concept of teleology in biology has been notably discussed by Ernst Mayr, who argued “teleonomy”—a term introduced by Colin Pittendrigh that suggests a union of lawfulness and purposiveness—to refer to apparently end-directed phenomena in biology; he legitimated the biological use of the term teleonomic, rather than teleologic, to denote a purely mechanistic purposiveness, not driven by intentional agency and not applicable to historical processes like evolution. This purposiveness is confined to processes and behaviours, rather than to the structure and organisation, which are governed by a program or code of information (Dresow and Love 2023). Mayr stated: «*The key word in my definition of "teleonomic" is the term "program" […] regardless of its definition, a program is (1) something material, and (2) exists prior to the initiation of the teleonomic process […] The term is taken from the language of information theory. A computer may act purposefully when given appropriate programmed instructions*». This is an example of a cybernetic approach in biology, which will be discussed below; for an in-depth discussion of Mayr’s teleonomy and cybernetics, readers can refer to (Vitale 2021). Today, teleological explanatory concepts are openly used in endosymbiotic theories and ecological systemic hypotheses, such as those proposed by Lynn Margulis and James Lovelock, as well as in epigenetics. In addition to the Gaia principle, an example comes from the extended evolutionary synthesis, which considers agential decisions that occur via physiological pathways through which organisms can teleologically affect their evolutionary trajectories (Lewis 2024).

Evolution concerns adaptations, and the dynamics of adaptation are central. The concept shifts from homeostasis, as the cornerstone of integrative physiology, to homeokinetics and homeodynamics (Yates 2008): metabolic networks of living systems are flexible and marginal, allowing adaptations. The paradigm of control loops that maintain bodily variables within target values (i.e., homeostasis) does not account for the dynamic and resilient nature of bodily systems (Bothe et al. 2023). The recent field of network physiology traces its roots to the recognition that «*human organism is an integrated network where complex physiological systems, each with its own regulatory mechanisms, continuously interact*» (Bashan et al. 2012).

Network physiology began with the mapping of dynamic interactions, in which networked interactions among entities that change over time give rise to global behaviours and emergent states (Ivanov 2021). Data-driven and concept-driven approaches, linked with adaptiveness, paved the way for network physiology: the science devoted to the analysis of the structure that links the components of a complex physiological system.

As a continuous alternation of stressors and coping, load and recovery, perturbations and adaptations, both transient, metastable and stable properties emerge from the physiological interconnectedness. *Id est*, biology generates variation, whose sources in the evolutionary process are not limited to genetics (Dickins 2023). Variations trigger responses. Here is the concept of “tinkering” in biology: the opportunistic rearrangement of elements (Laubichler 2007). Emergent phenomena in biological networks are high-level properties shaped by tinkering and not reducible to the properties of their components (Solé and Valverde 2020). Network concepts and models have been extended from biology to the entire health system, thereby encompassing socio-cultural systems. Interdependencies among environmental, socio-cultural, behavioural, molecular, organ, and cellular factors determine a plethora of states; hence, interacting components of a complex system give rise to novel behaviour that is not present in or predictable from its components (Sturmberg et al. 2019). It sounds akin to the Gestalt theory: «*the whole is something else than the sum of its parts, because summing up is a meaningless procedure, whereas the whole-part relationship is meaningful*» (Koffka 1935). As stated in the Introduction, the very foundations of the existence and use of network models in biology remain elusive. The following section will outline theories related to network biology that are based on relational and evolutionary concepts.

### A matter of relations and edges

Bizzarri and colleagues, arguing their mesoscopic way of thinking to deal with the epistemology of biology, affirmed «*A phenomenon can in principle be studied at different levels (sub-atomic, atomic, molecular, cell, *etc*.), but we are mostly interested in “effective” relationships, i.e., those relations triggering “emerging properties” of the system at a higher organization layer*». They advocated a re-thinking of the concept of biological causality through relational ontology by stating «*The basic idea of a relational ontology is that, in our inventory of the world, relations are somehow prior to the relata (i.e., entities*» (Bizzarri et al. 2019). Relational ontology does not imply the irrelevance of relata; instead, it rests on the fact that the same system of relations can be realised by different kinds of relata, thereby treating relations ontologically prior to relata. However, this raises a philosophical problem: how can the relation be *ontologically* prior to its relata? Bizzarri and colleagues state “somehow”, without further details and showing a certain degree of uncertainty. A similar problem arises with the so-called “ontic structural realism” (OSR), or *Relational Monism*, a Quantum Physics theory developed primarily by James Ladyman, Don Ross and Steven French, and endorsed also by Carlo Rovelli and N. David Mermin (Bondi 2024). The fundamental metaphysical principle at stake is conceived as a *structure of relations*, understood a priori with respect to any individual entity, and as that which most adequately accounts for the emergent properties of particles, such as spin entanglement. These properties, in fact, would not be properly “emergent”, rather the opposite: it is the very individuality of particles that emerges from the underlying, all-encompassing relational order. More broadly, this perspective recalls the *Gestalt Theory* (Amann 1993), or even the environmental ontology presupposed by deep ecology in the metaphysical sense articulated by Arne Naess (the so-called “Ecosophy T”). Yet every relational ontology faces a serious problem of coherence: the question of whether it is possible to speak of relation(s) without at the same time presupposing the existence of entities that enter into relations. One may nevertheless affirm that specific quantum properties, such as spin entanglement (a phenomenon where the spin of two or more particles, as a pair of electrons, remains linked regardless of the distance), are indeed emergent, insofar as they are irreducible to their constitutive parts (in this case, irreducible to the properties of individual electrons). In this sense, one is justified in speaking of *quantum holism*: entanglement can be taken as an almost paradigmatic instance of an “emergent property”. However, holism does not in itself entail the necessity of a relational ontology. It is possible to conceive of irreducible properties emerging from the relations between ontologically distinct entities. The same applies, *mutatis mutandis*, to the epistemology of biology. Rather than postulating a problematic relational ontology, one may instead endorse a holistic biology and a relational epistemology, according to which the most adequate way to describe living systems is to privilege the analysis of their internal and external relations, as well as of their emergent properties, without thereby positing relations as ontologically prior to entities that stand in relation.

Dickins in evolutionary biology has also highlighted the fundamental role of relationships. He followed the criticism of gene-centrism, arguing that the comprehensive instructional role attributed to genes came at the expense of other developmental processes, which are important for evolutionary dynamics. Then he argued that «*Information* […] *is not a thing, but a functional relationship* […] *between data and context, between input and system*» (Dickins 2023). Dickins’ viewpoint relies on evo-devo biology. This mechanistic approach, as argued by DiFrisco and Jaeger, explains evolutionary change as the modification of developmental mechanisms and processes, thereby overcoming the philosophical analysis focused on decomposition and functional localisation (DiFrisco and Jaeger 2019). Those two authors argued that, «*although “network thinking” of this sort represents a major improvement over classical gene-trait atomism, it still falls short of fulfilling the mechanistic research agenda of evo-devo.* […] *The proposed alternative is to integrate dynamical modeling of developmental processes into empirical practice alongside the identification of system components and their structural relations*».

Moving to psychology, Harry Heft, in his ecological approach rooted in Darwinian perspectives, argued that humans «*live in a functionally meaningful econiche that is constituted by the interdependent relations among living and nonliving entities*» that are «*dynamic, being marked by change as well as stabilities that are maintained for periods of time in the face of change*» (Heft 2013). Trying to overcome some criticism on Gibsonian ecological psychology regarding an almost brain-free model, Luis Favela has recently proposed his NeuroEcological Nexus Theory (NExT), which complements Gibsonian theory of “affordances” of environmental information with the theory of neural manifolds, whose systematic relationships give meaning to the neural, bodily, and environmental contributions to human behaviors and thoughts (Favela 2024). Favela’s theory is rooted in recent evidence from complex and network models, which have permeated neuroscience.

Are there any core threads across life and human science models that address variation, interdependent relationships, and evolution? The interconnected entities are connected to environment and evolution in Ervin Laszlo’s systems philosophy, i.e., a systems theory rooted in the natural scientific worldview, in which self-sustaining systems replace the concept of organisms in a changing natural environment. This theory is based on Whiteheadian naturalistic process philosophy and von Bertalanffy’s general systems theory. He developed his view, positing the inherent interconnectedness of systems (including those within the life sciences) that is fundamentally rooted in the information field associated with the quantum vacuum (Laszlo et al. 2011). In Laszlo’s viewpoint, everything is connected with every other thing, and events can be synchronised over great distances in the “psi-field”; this highly coherent and immortal world is organised into various levels of organisation, which influence each other, resulting in a network of networks where all the things leave their roots. In Laszlo’s conceptualisation of living systems, the information rooted in the psi-field persists beyond the demise of living elements, and the world as a whole can be construed as a dynamic, self-sustaining system.

The roots of interconnected entities that give rise to self-sustained systems are fundamental to the understanding of stigmergic communication, where communications between agents can be indirect and some information are shared through the environment; therefore, in stigmergic communication, interactions are the key, and information is crucial (Olsen 2019). Stigmergy, as a principle of spontaneous ordering or self-organisation, was first proposed by the entomologist Pierre-Paul Grassé in 1959 (Grassé 1959) and later shown to apply to complex systems beyond insect societies. The etymology derived from the Greek terms *stigma* and *ergon*, and Francis Heylighen moved from the Grasse ´’s interpretation, for which the product of work functions as a stimulus for action, to his own definition: «*stigmergy is an indirect, mediated mechanism of coordination between actions, in which the trace of an action left on a medium stimulates the performance of a subsequent action*». Stigmergy, «*even allows the coordination of ‘‘agentless’’ actions*» (Heylighen 2016). Heylighen’s background deals with cybernetics, systems theory, and complexity, arguing that the building blocks of reality are interactions, and systems are stabilized, self-producing networks of processes. In his evolutionary-cybernetic view, humanity can be conceived as a “Global Brain”. His model of stigmergy relies on the core components of action, medium, trace, spontaneous coordination, and feedback (Heylighen 2016). Within this framework, stigmergic coordination can be applied to a variety of aspects of plant behaviour: Sims and Yilmaz recently argued that, «*considering any organism that can coordinate sensing of the environment with motion within that environment is minimally cognitive […] plants in such situations are cognitive*»; thus defining a «*plausible conceptual framework for reconceiving plant cognition in distributed terms*» (Sims and Yilmaz 2023). In Sims’ view, «c*ognition is the coordination of multiple system processes through a trace variable that is itself the result of system actions on the world*» (Sims 2023).

Overall, current theories of stigmergy and their applications link network biology to relational and evolutionary concepts into a vision of biology which is firmly rooted in cybernetics. From the philosophical side, evolutionary-systemic philosophy and related theories endorse cybernetics as pivotal for biological philosophy. Biological models resulting from complex systems are essentially based on a) stimulus, b) response in the tuned refinement of traces, c) sensing mechanisms, d) actions, and e) emergent behaviours resulting from network interactions across spatiotemporal scales. However, the concepts of self-organisation, opportunistic rearrangement, and multiple systems resulting from actions through traces, grounded in minimal cognition, may even lead to the conceptualisation of a paradoxical “humanless humanity”. The fundamental issue pertaining to the nature of relations in biology is yet more problematic when linked to concepts of organism, human being, and person. A cybernetic organism (*cyb-org*) is not necessarily a cybernetic person (*cyb-per*). How can concepts such as distributed cognition and agentless actions imply the presence of persons? Are the biological models derived or at least grounded on network science paving the way for cybernetic theories of the person?

### Philosophical arguments over network biology, cybernetics and relational models

If we recall the first meaning of the term “cybernetics”, its use to refer to human beings, far from being problematic, is convincing and even obvious. The term “cybernetics” was coined by the American mathematician-philosopher Norbert Wiener from the Greek word “kubernetes”, meaning steersman or helmsman (see Sachs 2022). Thus, when applied to a goal-oriented system, it appears to be nothing more than another anthropomorphic metaphor; when applied to human beings, it seems pretty natural. Human beings are archetypal “cyborgs”: goal-oriented systems that learn primarily through feedback.

The situation becomes more problematic, however, when we introduce the phenomenon of consciousness: a steersman is aware of the destination towards which he is steering the boat; this includes also that he can change this destination not only due to cybernetic mechanisms of trials, errors, and adjustments, but simply because they *want to*. This is the core point of the philosophical concept of “person”, as distinct from “human being”. A person is a cyborg that can act in non-cybernetic ways precisely because they know they are fundamentally a cyborg.

This point can be clarified by considering the potential replacement of all teleological/teleonomic explanatory models with cybernetic ones. The famous psychologist Gregory Bateson notably expressed his “theoretical enthusiasm” about the recursive nature introduced by feedback mechanisms within cybernetic processes, whether biological or digital ones: «It was a solution to the problem of purpose. Since Aristotle, the final cause has always been a mystery. This came out then. We didn’t realise then [in the early 40 s] that the whole of logic would have been reconstructed for recursiveness» (Brand 1976). Actually, this statement is not entirely grounded: cybernetic circularity—where the effects of a system’s outputs feed back as inputs, influencing its future state and actions—can explain the functioning, the evolution and even the self-learning ability of a system, but it doesn’t imply the disappearance of a purpose which orients the whole process. This purpose can be internal (as in a living organism that is genetically programmed to replicate itself) or external (as in a thermostat set by a human). It is no coincidence that the father of cybernetics, Norbert Wiener, contrary to Bateson and precisely in those same 40 s, used for one of the very first times the term «feed-back» (with the hyphen) as a synonym of «teleological purposeful active behavior» of a system (Rosenblueth et al. 1943). Nevertheless, a significant distinction exists between acting without awareness and acting with an understanding of the underlying or overarching purpose of our actions. Currently, this level of consciousness—which is essential for enabling individuals to set new or alternative purposes—appears to remain unique to human beings, thereby distinguishing them as “persons”. Andrews highlighted how certain animals also exhibit various degrees of properties that are constitutive of personhood, such as sentience, emotions, autonomy, self-awareness, sociality, language, rationality, narrative self-constitution, morality, and meaning-making, thereby raising ethical considerations (Andrews 2020). However, the debate on animal consciousness and personhood extends beyond the aims of this work. The origin of consciousness remains a major theoretical and scientific issue (Seth and Bayne 2022). In any case, it seems that the perception of the external world as something “external” is central to the formation of the idea of self, viz., to self-awareness. Without relation, there would be no human person. This is true both for internal and external relations. Philosophically speaking, we can highlight several relational elements which contribute to the constitution of the human person: the social environment in which a person grows up, the perceptions which enable the reflection, the body (and especially the face) as a threshold between interiority and exteriority, the more or less mediated interpersonal relations, the internal dialogue which often happens when we think about life events or choices to make. Physiologically speaking, the relational elements which contribute to define a human person can be identified as interacting molecules, cells, tissues, organs and systems that allow bodily functions, and on consciousness itself, whose neural correlates rely on brain networks [see e.g. (Koch et al. 2016) and (Duclos et al. 2021)]; sensing, elaboration, responses and adaptations are inherently linked to relational processes that connect a person to other living beings and to the environment.

The very concept of “person” has a history in philosophy and theology [see (Bondi 2023)], in which the role of relation is central. Considering the relation as at least a necessary element of a person, the classical notion of individual essence has to be enlarged, embracing something between the immutable substance and the mutable accident: Simplicius, for example, wrote that «*among the [Aristotelian] categories, it is only characteristic of the relation (pros ti) that one exists in many entities*» (Simplicius 1907). This means that relational entities must also be considered in what they share with—and/or communicate to—other entities, thus potentially creating a dynamic cybernetic system. In the end, without falling into a theoretically problematic relational personalistic ontology, according to which the person would be *nothing else than their relations*, we can properly consider the person as a “relational cybernetic organism”, with the addition that consciousness constitutes the pole in which the different (internal and external) relations are experienced, thus configuring a *further* relationship. The latter allows the human person to reorient the goals of their own cybernetic system.

In philosophical biology, fundamental questions concerning the nature of relations are also posed regarding the determination of biological identity. Pradeu has expressed criticism of the utilisation of the category of “organism”, which is defined as a functionally integrated and cohesive whole made of interdependent and interconnected parts, as being equivalent to and interchangeable with the concept of “biological individual”. He argued that this definition of “organism” should be applied to “physiological individual”. In contrast, “evolutionary individual”, which also refers to a “biological individual”, can be defined in terms of a unit, either reproductive or selective (Pradeu 2016a). In philosophical debates about biological individuality, he highlighted objections related to monism, theory-centrism, ahistoricity, disciplinary isolationism, and conceptual uncertainties, thereby calling for a more unified definition inspired by historical and metaphysical considerations that also suits current biological perspectives and practices (Pradeu 2016b). We have argued that the operationalisation of network models in living entities may give rise to philosophical postures that are prone to cybernetics and relational personalistic ontologies. To address the issues raised in this article regarding living entities and network models and theories in the philosophy of biology, we propose the philosophical stance of “epistemic humility”.

## A call for epistemic humility

Since the paradigm shift in physics in the last Century, scientists, researchers, and philosophers have begun to adopt the idea of epistemic humility, which was later formalised (Matthews 2006; Van Cleve 2011). By “epistemic humility”, we mean becoming aware of the inevitable limits of scientific knowledge, without rejecting realism or epistemological criteria, but certainly recognising that the possibility of achieving a complete, *objective* scientific understanding of the world is nothing more than a myth of modern science, especially in physics before quantum mechanics. This myth was based on the presumptuous illusion of being able to view the world “from God’s point of view”, as if the observer could remove themselves from the equation of the world and could thus attain knowledge of the thing-in-itself. This is even more evident if we refer to relational biological models in which we ourselves, as human beings, are “nodes in the net”. We should clarify, here, that not being able to know something “in itself” does not in any way imply that nothing exists independently of our knowledge or perception. To stick with Einstein’s example (Pais 1979), the fact that something manifests to our understanding and senses as “the moon” is not undermined by the fact that what I call “moon” does not correspond precisely to that thing. Without the idea of “nature” —that is, the idea that something exists independently of our will—science itself would be impossible. For example, the surprise of the first quantum physicists at the strangeness of the results of their experiments cannot be explained as anything other than wonder at something that exists independently of me and my expectations and knowledge. If something unexpected arrives, it is because it exists independently of the person who expects something else. As Heisenberg summarises, «*natural science does not simply describe and explain nature; it is part of the interplay between nature and ourselves; it describes nature as exposed to our method of questioning*» (Heisenberg 1962, p.81). Ontological realism and epistemic humility can together encourage scientists to investigate what nature reveals to their inquiring knowledge. In other words, they enable scientists to explore how nature probably is, in accordance with the epistemological criteria of their disciplines.

Despite the remarkable success in describing diverse systems across biology, network models still require development of more conceptually expansive frameworks to capture the contextual, multilevel, and adaptive nature of biological systems; Pessoa defines this issue an epistemological opportunity to build models that evolve with context dependence, open-endedness, and history sensitivity (Pessoa 2026). In their epistemic model of network science focused on the possibility of taming vagueness properties offered by network models, Elek and Babarczy highlight to possible postures for explaining phenomena related to mechanisms of complex systems: the one favouring the actual and concrete and the other favouring the theoretical and the universal (Elek and Babarczy 2022). The philosophical posture of epistemic humility admits the open-ended nature and vagueness properties of complex systems thus enabling scholars to investigate what relational biological models reveal and what probably represent accordingly to epistemological criteria. More deeply, a posture of epistemic humility seems to be necessary when analysing the history of science and the historical development of scientific perspectives on the world: relational models themselves, in a historical (and relational!) way, exhibit a link with particular cultural phenomena that do not fall within the domain of science.

## The network is a prominent contemporary *meme*

It is worth noting that during the modern age, with the significant progress of technology and mechanics, nature was thought of as a deterministic mechanism (a “clock”), and living beings as fighting each other to survive (as they were bourgeois who want to enrich themselves at the expense of others); while in our era, where sharing and information technology dominate the cultural scenario, the mainstream metaphor is precisely that of the network. It is therefore our intention to define the network as a contemporary *meme* that has spread and permeated various fields of knowledge and research.

The sociologist Manuel Castells intensely studied the “network society”, stating: «*as an historical trend, dominant functions and processes in the Information Age are increasingly organized around networks*» (Castells 2010). In network society, the network is a standard method, form and metaphor. Technical networks surround us, and networking and interactivity dominate contemporary thinking on individual and collective life. In the second half of the twentieth century, early network ideas within psychology and anthropology developed into social science (Knox et al. 2006). Network has been used as a generic label embracing different types of state/society relations in policy (Börzel 2011). More generally, the network serves as a powerful metaphor even without a specific theory to support it, as it enables the unification of apparently disparate elements (Jones 2004).

In this particular scenario, the field of network biology was nurtured by network scientists, in conjunction with bioinformaticians and clinicians. The development of network physiology was underpinned by the perspectives and models proposed by physicists, mathematicians and bioengineers. Modern physiology has nourished the concept of networks, as living entities can be conceptualised as a plurality of bodily components that interact with one another and are regulated in response to variation (Pradeu 2016a). A conceptualisation of network models in biology can be found in complex adaptive systems, where interactions among agents give rise to emergent properties and behaviours. The properties of biological entities as complex adaptive systems give rise to emergent phenomena at higher levels, and their interactions with other biological entities have been modelled as dynamic, self-sustaining systems within networks of networks. Consequently, the contemporary meme that pervades the Information Age has been paving an ever-changing avenue for biological theories and applications.

**Fig. 1:**
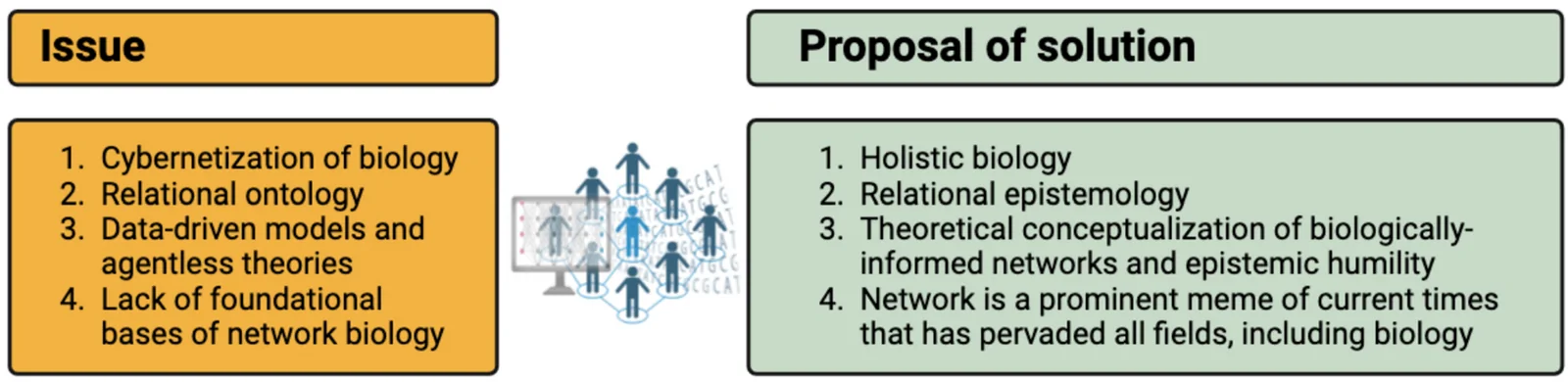
List of issues related to network models in biology and philosophical possible solutions. Image created with BioRender (https://www.biorender.com/)

## Conclusions

Networks have permeated every aspect of human life, including biology, and continue to expand rapidly. Here, we argued for the possibility of philosophically endorsing a holistic, rather than cybernetic, biology. We also argued the philosophical endorsement of relational epistemology, rather than relational ontology. Living systems exist and can be described by focusing on their internal and external relationships and their emergent properties. Within this viewpoint, network models are a great weapon at our disposal. We defined a foundational issue in network models in biology by merging bioinformatics outbreak with the sharing approach of recent decades, thereby elevating network as a prominent social metaphor that pervades, among other fields, biology (Fig. 1).

According to ecological psychology and ancillary, not brain-free, models, influences rooted in sociocultural history have shaped human development. At the same time, systemic relationships among neural manifolds give rise to human higher functions. According to systemic philosophy, organisms are defined as self-sustaining systems that are intrinsically interconnected and distinguished from the changing natural environment. The prevailing threads across biological models about interdependent relationships appear to be grounded in the conceptualisation of these relationships, thereby leaving minimal space for the person, who consequently becomes an agent within the network. The rise of systems biology, with its emphasis on data-driven models, may have contributed to this conceptualisation. Applications of data-driven models often provide limited transparency and explainability, which continue to constrain their deployment in biomedical settings. Solutions have already been proposed, focusing on biologically informed models, explainable artificial intelligence (XAI), and biological interpretability (Wysocka et al. 2023). In particular, a theoretical generalisation of biologically informed networks that integrates prior biological knowledge into existing AI frameworks for network modelling will constitute a foundational reference.

The concepts of minimal cognition, tinkering, and stigmergy may be considered pivotal yet insufficient for describing the person. In fact, these premises would already be applicable to define even a plant system as a person. The definitions of distributed cognition and agentless actions, possibly modelled through multilayer network interactions, are not sufficient to philosophically endorse a humanless humanity and a *cyb-per*. Instead, we argued for the philosophical endorsement of the person as a “relational cybernetic organism”.

Without a theoretical conceptualisation, data-driven models that are revolutionising biology risk drifting into sensationalism or producing an overdose of data that cannot be successfully interpreted and applied, leading to the cybernetisation of the biological sciences. As recently argued by DiFrisco and Orzack, the practice of philosophers into biology should contribute to useful clarification of concepts and development of theories, while biologists should accept the essential role of concepts in their understanding of nature and even incorporate the concept-based understanding into research practice (DiFrisco and Orzack 2026). We propose elevating epistemic humility to mitigate the aforementioned risk by utilising a relational standpoint to define the foundations of relational models.

## Data Availability

No datasets were generated or analysed during the current study.
